# Innate immune sensing of danger signals: novel mechanism of heme complex-mediated lytic cell death

**DOI:** 10.1038/s41392-024-01985-z

**Published:** 2024-09-30

**Authors:** Chintan Chhatbar, Michael Schulz, Roman Sankowski

**Affiliations:** Institute of Neuropathology, Faculty of Medicine, University of Freiburg, Freiburg, Germany

**Keywords:** Innate immunity, Innate immune cells

In their recent publication in *Cell*, Sundaram, and colleagues identified a novel role for NOD-like receptor (NLR) family CARD domain containing 5 (NLRC5) in heme-mediated cell death and inflammation.^1^ This process is initiated by Toll-like receptors 2 and 4 on the cell surface, which induces intracellular NAD+ depletion and ROS production.

The NLRs represent a large family of immune sensor proteins. Most of its members characterized so far were shown to induce pyroptosis. The most extensively profiled member of the NLR family, NLR pyrin domain-containing (NLRP) 3, is an integral component of “inflammasomes” which mediate inflammatory cell death. Prior to the study, NLRC5 remained among the least understood members of this group of proteins. Earlier studies have shown that NLRC5 functions as an MHC class I transactivator (CITA), regulating MHC class I gene expression.^2^ This regulation is driven by the translocation of NLRC5 between the nucleus and cytoplasm, a process controlled by interferon-gamma (IFN-γ) and critical for T cell function. T cells exhibit high levels of NLRC5 expression. Beyond that, the function of NLRC5 as a sensor of danger signals and its function in the innate immune response remains unclear.

**Fig. 1:**
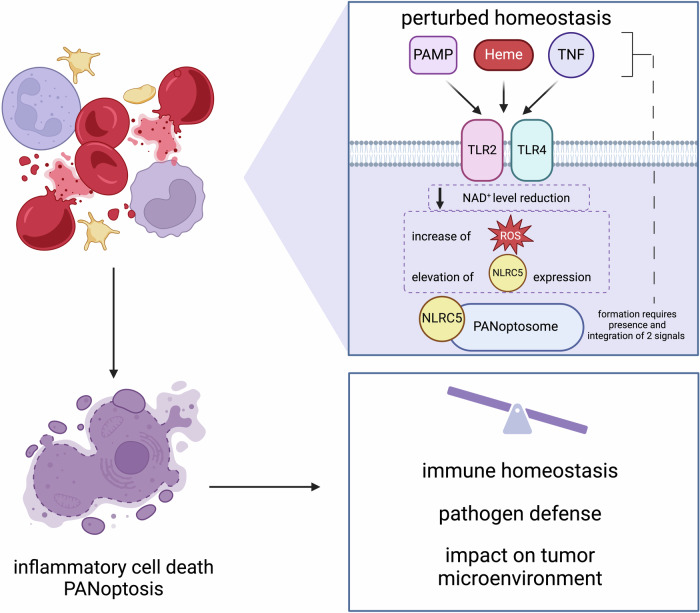
NLRC5 functions as an innate immune sensor to regulate PANoptosome formation and immune cell death. Sensing of heme together along with a secondary signal derived from either cytokines (e.g., TNF) or pathogen-associated molecular patterns (PAMPs, e.g., LPS) induces intracellular NAD+ reduction, increased ROS, and NLRC5 expression. Ultimately, this leads to the induction of inflammatory cell death, PANoptosis, in macrophages via PANoptosome formation, which includes NLRC5 and other proteins. The consequences for immune homeostasis, inflammatory responses, and the tumor microenvironment remain to be explored. The figure was adapted from the graphic abstract of the original study^1^ and created with BioRender.com

To address this knowledge gap, Sundaram et al. conducted an unbiased screen for potential NLRC5 ligands using various pathogen-associated molecular patterns (PAMPs), damage-associated molecular patterns (DAMPs), and cytokines, both individually and in combination. They assessed lytic cell death in bone marrow-derived macrophages (BMDMs). The study found that NLRC5 is essential for inducing inflammatory cell death in response to heme combined with additional PAMPs (e.g., LPS) or TNF. This process, called PANoptosis, involves the PANoptosome complex, which includes NLRC5, NLRP12, apoptosis-associated speck-like protein containing a caspase activation and recruitment domain (ASC), receptor-interacting protein kinase 3 (RIPK3), caspase-8, and NLRP3. Notably, the authors show that NLRC5 does not activate caspase-1 and NLRP3 was not required for the formation of the complex. However, in the absence of NLRC5, NLRP12, or ASC, PANoptosome complex formation was impaired. Heme sensing began at the cell surface through Toll-like receptors (TLR) 2 and 4, and NLRC5 expression was induced by NAD+ depletion. Inflammatory cell death was mediated by NLRC5 upregulation and reactive oxygen species (ROS) production. In summary, the study highlights the disease relevance of NLRC5, showing its role in heme-mediated kidney damage as well as inflammation and mortality in hemolytic disease, hemophagocytic lymphohistiocytosis (HLH), and colitis. These results suggest NLRC5 as a potential therapeutic target for inflammatory diseases involving PANoptosis (Fig. 1). Given the acute nature of the cell lytic process, a role of T cells appears unlikely, supporting the notion of a truly novel function of NLRC5 in innate immune cells.

Despite extensive experimental validation, the authors acknowledge several limitations of their study. For instance, the interaction of NLRC5 and NLRP12 appears surprising due to the lack of an association domain and needs further characterization. A potential interaction at the protein level might be possible via the NACHT domain, although these sequences in the middle of the proteins are phylogenetically distant. Additionally, since previous studies have not demonstrated direct NLRC5 induction by TLR ligands, the mechanism of its induction by the combination of Pam3CSK4 and heme needs to be clarified. Given the known involvement of IFN-β and IFN-γ in NLRC5-related immune processes, these cytokines may play a role in the signaling cascade leading to NLRC5 induction and heme-induced cell death.

From a translational perspective towards novel clinical management strategies of hemolytic disorders, a detailed mechanistic understanding of the role of NAD+ is also crucial going forward. These and other open questions present opportunities for follow-up studies. The findings underscore heme’s importance in inflammatory cell death and raise questions about its pivotal role requiring dedicated receptors.

Like other DAMPs, heme is an evolutionarily conserved danger signal recognized by specific invariant receptor proteins. Binding of DAMPs triggers a rapid immune response, bridging the time before an adaptive response can be mounted. Among different DAMPs identified in recent years, heme is particularly well studied. This is due to its ubiquitous presence among mammalian, bacterial, and plant cells. With porphyrin rings as basic components, heme has evolved as an electron transport agent involved in metabolic processes. In mammals, it is tightly packed with hemoglobin. During cell damage events like hemolysis, extracellular heme is cytotoxic and must be quickly removed to limit damage. Sundaram et al. identify the evolutionary conserved NLRC5-NLRP12 axis as a key player in heme-induced inflammation and tissue damage through PANoptosis, demonstrated using multiple disease models in NLRC5-knockout mice.

Throughout life, elevated heme levels occur in both physiological and pathological situations. For example, physiologically high levels of the heme degradation product bilirubin lead to neonatal jaundice, while pathologically, intravascular hemolysis can lead to acute kidney failure due to excessive heme in the blood. Intravascular hemolysis can result from various conditions, including hereditary hemolytic anemias, autoimmune hemolysis, trauma, and infections. The authors, using public datasets, demonstrate that malaria infection, linked to sickle cell disease, induces NLRC5 expression in monocytes, highlighting their critical role in responding to elevated heme levels.^1^ Monocytes both trigger inflammation to support anti-parasitic immunity in an NLRC5-dependent manner and mediate heme clearance to prevent secondary organ damage.

Monocyte-driven heme clearance is one of several protective mechanisms against heme toxicity. Notably, heme is one of the few known factors that can independently shape macrophage identity. It induces the transcription factor SPI-C, driving blood monocytes to differentiate into iron-recycling macrophages.^3^ Monocytes sense baseline heme levels through the high-affinity hemoglobin-haptoglobin complex receptor CD163, which is highly expressed on monocytes and certain tissue-resident macrophages, including perivascular brain macrophages.^4^ CD163 regulates cerebral blood flow by sampling soluble blood components.^5^ Notably, CD163 was upregulated in monocytes that infiltrate the brain under certain conditions.^4^ In glioblastoma, monocyte-derived macrophages continuously engraft and maintain high CD163 levels, while glioblastoma-associated microglia also increase CD163 expression. This high expression, which is linked to an anti-inflammatory M2-like macrophage phenotype, has been associated with poor prognosis in glioma patients in association studies. One possible explanation is that CD163 scavenges hemoglobin-haptoglobin complexes, lowering heme levels, thereby possibly reducing NLRC5 expression, contributing to T cell exhaustion in glioblastoma.

Although it is not yet possible to induce NLRC5 expression pharmacologically, its activation could offer a promising therapeutic strategy to enhance immunity against glioblastoma and other cancers. This could lead to the identification of novel immune checkpoint molecules, bridging innate and adaptive immunity, similar to targeting mutant isocitrate dehydrogenase 1 (IDH1) in a subset of gliomas.

In conclusion, the newly identified role of NLRC5 in heme-complex-mediated PANoptosis opens new pathways for immune activation in previously untreatable conditions. These conditions may turn out to be as diverse as autoimmunity, infection and malignant diseases.
